# Phase I and pharmacokinetic study of trabectedin, a DNA minor groove binder, administered as a 24-h continuous infusion in Japanese patients with soft tissue sarcoma

**DOI:** 10.1007/s10637-014-0094-5

**Published:** 2014-04-03

**Authors:** Takafumi Ueda, Shigeki Kakunaga, Masashi Ando, Kan Yonemori, Hideshi Sugiura, Kenji Yamada, Akira Kawai

**Affiliations:** 1 Department of Orthopaedic Surgery, Osaka National Hospital, 2-1-14 Hoenzaka, Chuo-ku, Osaka, 540-0006 Japan; 2 Breast and Medical Oncology Division, National Cancer Center Hospital, Tokyo, Japan; 3 Department of Orthopedics, Aichi Cancer Center Hospital, Nagoya, Aichi Japan; 4 Division of Musculoskeletal Oncology, National Cancer Center Hospital, Tokyo, Japan

**Keywords:** Pharmacokinetics, Clinical trial phase I, Soft tissue sarcoma, Trabectedin, Chromosomal translocation

## Abstract

*Background* Trabectedin is a novel anticancer agent used to treat soft tissue sarcoma (STS). This phase I study of trabectedin was performed to determine the recommended dose for phase II studies in Japanese patients with STS. *Methods* Patients who had STS refractory to, or who could not tolerate, anthracycline-based chemotherapy were enrolled. The starting dose of trabectedin was 0.9 mg/m^2^, given as a 24-h continuous infusion every 21 days. The dose was escalated to 1.2 mg/m^2^ and then to 1.5 mg/m^2^, using a “3 + 3” cohort expansion design. Plasma samples were collected for pharmacokinetic analysis. *Results* Fifteen patients received 1 of 3 dose levels of trabectedin. Dose-limiting toxicity occurred in two of three patients at 1.5 mg/m^2^: 1 had a grade 3 increase in creatine phosphokinase and grade 3 anorexia, and the other had grade 4 platelet count decreased. Frequent grade 3 or 4 adverse events (AEs) included elevations of alanine aminotransferase and aspartate aminotransferase and decrease in neutrophil count. The frequency and severity of AEs were clearly greater at 1.5 mg/m^2^ than at the lower doses. Pharmacokinetic analysis showed that the area under the concentration-time curve at a dose of 1.2 mg/m^2^ was adequate to produce antitumor activity. A partial response was obtained in three patients with translocation-related sarcomas (1 each with myxoid liposarcoma, synovial sarcoma, and extraskeletal Ewing sarcoma). *Conclusions* The recommended dose of trabectedin for phase II studies is 1.2 mg/m^2^ in Japanese patients with STS. Trabectedin may be especially effective against translocation-related sarcomas.

## Introduction

Soft tissue sarcomas (STS) are a heterogeneous group of rare malignant tumors of mesenchymal origin that account for less than 1 % of all adult malignancies. Chromosomal translocations are the most frequent molecular alterations in sarcomas, occurring in about 20 % of cases [1]. Sarcoma translocations and the associated chimeric oncoproteins provide attractive targets for therapeutic intervention, given that these fusion proteins are critical for disease pathogenesis and tumor-cell survival, and no alternative pathways exist to avoid their blockade [2–5].

Current treatment options for patients with STS vary according to clinical stage, but include surgery, radiotherapy, and chemotherapy [6]. As for chemotherapy, doxorubicin and ifosfamide, given sequentially as single agents or in combination, have been used as standard treatment for most histologic subtypes of advanced STS to date [7], however, the outcomes of patients with advanced or metastatic sarcoma remain poor over the past two decades, with an estimated median survival of approximately 1 year from the start of first-line therapy [8–11]. Recently, pazopanib, a multitargeted tyrosine kinase inhibitor has demonstrated single-agent activity in patients with advanced STS subtypes, excluding liposarcomas, in a phase III trial [12]. Several guidelines have included pazopanib and other chemotherapy as options for palliative therapy [13, 14], but there is a paucity of high-level evidence to support.

Trabectedin is a tris tetrahydroisoquinoline alkaloid initially isolated from the marine ascidian, *Ecteinascidia turbinata*, and is now produced synthetically. This agent binds to the minor groove of DNA and interacts with proteins of the DNA repair machinery, disrupting the cell cycle and inhibiting cell proliferation [15]. Trabectedin has been approved in the European Union and other countries worldwide, with the exception of the United States and Japan, for the treatment of advanced STS after failure of anthracycline and ifosfamide. Some clinical guidelines recommend trabectedin as a second-line option [13, 16, 17].

Pharmacokinetic studies of trabectedin administered as 24-h continuous infusion in patients with solid tumor showed linearity within the dose range studied (0.05–1.8 mg/m^2^), with large inter-patient variability and moderate intra-patient variability [18]. A population pharmacokinetic (PopPK) analysis [19] derived from 603 cancer patients who received single-agent trabectedin concluded that none of the subject covariates were significantly related to between- or within-subject variability in the plasma clearance of trabectedin. Although trabectedin is considered to have a narrow therapeutic index, evidence suggesting ethnic differences in the safety and tolerability of trabectedin remains scant. Moreover, clinical trials of trabectedin have been conducted mainly in the Caucasian patients and there is little data of trabectedin for Asian patients.

This was a phase I pharmacokinetic study of trabectedin in Japanese patients with advanced STS. The primary objective was to determine the recommended dose of trabectedin for phase II studies in Japan.

## Patients and methods

### Patient eligibility

Patients were eligible if they were 18 years or older with an Eastern Cooperative Oncology Group (ECOG) performance status (PS) of 0 or 1, and had a histologically confirmed diagnosis of STS and had received at least one anthracycline-based regimen and a maximum of up to four previous lines of systemic therapy for advanced disease. Hematologic, hepatic and renal function had to be confirmed based on laboratory assessment.

Patients were excluded if they had received surgery during the 4 weeks before study entry; radiotherapy or chemotherapy during the 3 weeks before study entry. Pregnant or breast-feeding women were also ineligible, as were patients who had any of the following conditions: severe complications; symptomatic brain metastasis; a history of neoplasms; pleural effusion, ascites, or pericardial fluid requiring drainage.

The study was conducted in accordance with the International Conference of Harmonization guideline for Good Clinical Practice and with the Declaration of Helsinki. The protocol was approved by an independent review board at each investigational site, and written informed consent was obtained from all patients before enrollment.

### Treatment plan

Trabectedin was supplied by Taiho Pharmaceutical Co., Ltd. (Tokyo, Japan) as a lyophilized powder in glass vials. The drug was administered as a 24-h continuous intravenous infusion via a central venous access catheter. Cycles were repeated every 3 weeks until disease progression, unacceptable toxicity, or withdrawal of consent. The starting dose was 0.9 mg/m^2^, which is equivalent to half of the maximum tolerated dose (MTD; 1.8 mg/m^2^) in a previous phase I study of solid tumors [18] conducted in France. The maximum dose level was 1.5 mg/m^2^, which is the approved dose in the European Union; a 1.2 mg/m^2^ dose was also evaluated as the midway point between 0.9 and 1.5 mg/m^2^. Dose escalation followed a 3 + 3 design [20]. The MTD was defined as the minimum dose at which more than 33 % patients had dose-limiting toxicity (DLT). The recommended dose for phase II clinical trials was defined as the dose level below the MTD.

### Assessments

Data on demographic characteristics and medical history were collected during screening. Physical examination and vital sign and safety assessments (PS, 12-lead electrocardiography, and laboratory test) were conducted at baseline/screening and throughout treatment.

### Safety

Adverse events (AEs) were graded according to the National Cancer Institute Common Toxicity Criteria (version 4.0). DLT was defined as any of the following: grade 3 decrease in platelet count requiring platelet transfusion; grade 4 decrease in platelet count; grade 4 decrease in neutrophil count lasting more than 5 days; febrile neutropenia; elevations of alanine aminotransferase (ALT) or aspartate aminotransferase (AST) to more than 5 times the upper limit of normal that do not recover to 2.5 times the upper limit of normal by day 28; any nonhematologic toxicity of grade ≥3.

### Tumor response

Tumor response was assessed according to the Response Evaluation Criteria in Solid Tumors guidelines (version 1.1) by the investigators at screening, every 6 weeks until week 24, and every 9 weeks thereafter.

### Pharmacokinetic studies

All patients underwent plasma pharmacokinetic studies between day 1 and day 8 of the first cycle. Heparinized whole blood samples were collected before the start of infusion; 0.5 and 1.5 h after the start of infusion; immediately before the end of infusion; and 0.5, 1, 2, 5, 8, 24, 48, 72, and 144 h after the end of infusion. Plasma concentrations were measured using a miniaturized liquid chromatography/tandem mass spectrometry method as described elsewhere [21]. Pharmacokinetic variables were calculated by non-compartmental analysis methods. C_max_ was obtained directly from pharmacokinetic data. The AUC up to the last observed time (AUC_0-t_) was calculated with the trapezoidal rule. The AUC_0-inf_ was calculated as the sum of AUC_0-t_ and the extrapolated AUC, calculated from the terminal rate constant λz (C_last_/λz, where C_last_ is the last measured concentration). The elimination half-life (t_l/2_) was calculated as 0.693/λz, and the total plasma clearance (CL) was calculated as the dose divided by the AUC_0-inf_. The volume of distribution at steady state (V_dss_) was calculated by the formula CL × AUMC_0-inf_/AUC_0-inf_, in which AUMC_0-inf_ is the area under the first-order moment curve.

### Statistical analyses

Sample size was determined empirically, based upon a 3 + 3 escalation design. Descriptive statistics were used for analyses of safety, tumor response, and pharmacokinetic variables. Safety was analyzed on the basis of data for the first 4 cycles. The data cutoff point for efficacy analysis was the end of July 2013.

## Results

### Patient characteristics and doses administered

Between September 2010 and September 2011, a total of 15 patients received 1 of the 3 dose levels. Patient characteristics are shown according to dose level in Table 1. As for histological type of sarcoma, fusion gene was confirmed in five patients (three patients with synovial sarcoma and 1 each with extraskeletal Ewing sarcoma and myxoid liposarcoma),

**Table 1:** Patient characteristics

		Level 1 (*N* = 3)		Level 2 (*N* = 9)		Level 3 (*N* = 3)		Total (*N* = 15)	
		n	(%)	n	(%)	n	(%)	n	(%)
Gender	Male	1	(33.3)	5	(55.6)	2	(66.7)	8	(53.3)
	Female	2	(66.7)	4	(44.4)	1	(33.3)	7	(46.7)
Age (years)	Mean (SD) Median	53.3 (11.7) 58		37.8 (13.5) 33		52.7 (11.7) 55		43.9 (14.3) 40	
Height (cm)	Mean (SD) Median	155.8 (8.9) 155.5		166.8 (8.4) 169.0		165.2 (6.1) 168.5		164.3 (8.8) 164.8	
Weight (kg)	Mean (SD) Median	56.0 (5.5) 57.1		62.4 (8.7) 56.5		66.0 (11.1) 68.8		61.8 (8.7) 57.1	
BSA (m^2^)	Mean (SD) Median	1.544 (0.128) 1.555		1.696 (0.138) 1.718		1.726 (0.169) 1.788		1.672 (0.148) 1.666	
PS	0	3	(100.0)	9	(100.0)	2	(66.7)	14	(93.3)
	1	0	(0.0)	0	(0.0)	1	(33.3)	1	(6.7)
Histological type	Leiomyosarcoma	2	(66.7)	2	(22.2)	1	(33.3)	5	(33.3)
	Synovial sarocma	0	(0.0)	2	(22.2)	1	(33.3)	3	(20.0)
	Extraskeletal Ewing tumor	0	(0.0)	1	(11.1)	0	(0.0)	1	(6.7)
	Dediffrenciated liposarcoma	0	(0.0)	2	(22.2)	0	(0.0)	2	(13.3)
	Myxoid liposarcoma	0	(0.0)	1	(11.1)	0	(0.0)	1	(6.7)
	Solitary fibrous tumor	1	(33.3)	0	(0.0)	0	(0.0)	1	(6.7)
	Spindle cell sarcoma	0	(0.0)	0	(0.0)	1	(33.3)	1	(6.7)
	Alvolar soft part sarcoma	0	(0.0)	1	(11.1)	0	(0.0)	1	(6.7)
Primary Lesion	Upper extremity	0	(0.0)	1	(11.1)	0	(0.0)	1	(6.7)
	Lower extremity	0	(0.0)	1	(11.1)	1	(33.3)	2	(13.3)
	Face	1	(33.3)	0	(0.0)	0	(0.0)	1	(6.7)
	Intrathoracic	0	(0.0)	1	(11.1)	0	(0.0)	1	(6.7)
	Chest, other	0	(0.0)	1	(11.1)	0	(0.0)	1	(6.7)
	Retroperitoneal	0	(0.0)	4	(44.4)	0	(0.0)	4	(26.7)
	Uterus	2	(66.7)	0	(0.0)	1	(33.3)	3	(20.0)
	Abdomen/pelvis, other	0	(0.0)	1	(11.1)	1	(33.3)	2	(13.3)
Location of lesions at baseline^a^	Lung	1	(33.3)	3	(33.3)	0	(0.0)	4	(26.7)
	Liver	0	(0.0)	2	(22.2)	0	(0.0)	2	(13.3)
	Lymph node	0	(0.0)	0	(0.0)	1	(33.3)	1	(6.7)
	Soft tisuue	1	(33.3)	3	(33.3)	0	(0.0)	4	(26.7)
	Other	1	(33.3)	3	(33.3)	1	(33.3)	5	(33.3)
Number of prior lines for advanced therapy	Median	2.0		2.0		2.0		2.0	
	Range [Min, Max]	[2, 3]		[1, 10]		[1, 4]		[1, 10]	

Analysis Set: FAS

*SD* standard deviation, *PS* performance status

^a^Multiple answers allowed

The median number of treatment cycles was 4, 4, and 2 at dose level 1, 2, and 3, respectively.

### Dose-limiting toxicity

DLT occurred in two patients at dose level 3. One patient had creatine phosphokinase (CPK) increased (grade 3) and anorexia (grade 3), and the other had platelet count decreased (grade 4). Both of these patients also had severe elevations of ALT and AST (>2,000 IU/L) with no clinically significant abnormalities of bilirubin or alkaline phosphatase and no sign of hepatic impairment. These events resolved 7 to 12 days after onset and did not meet the criteria for DLT.

### Toxicity

The incidence rates of adverse drug reactions occurring in three or more patients are shown according to dose level and grade in Table 2. The incidence rate and severity of AEs related to hepatic and bone-marrow toxicity increased in a dose-related fashion. At dose level 2, a patient had a grade 4 increase in CPK, but recovered from the event after delaying initiation of the next cycle. At dose level 3, all three patients had severe increases in CPK, and rhabdomyolysis was diagnosed in 1 of these patients.

**Table 2:** Incidence rates of adverse drug reactions occurring in 3 or more patients

Preferred term	Level 1 (*N* = 3)				Level 2 (*N* = 9)				Level 3 (*N* = 3)				Total (*N* = 15)
	G1	G2	G3	G4	G1	G2	G3	G4	G1	G2	G3	G4	Incidence rate^a^
	n (%)	n (%)	n (%)	n (%)	n (%)	n (%)	n (%)	n (%)	n (%)	n (%)	n (%)	n (%)	n (%)
Nausea	2 (66.7)				8 (88.9)	1 (11.1)			1 (33.3)	1 (33.3)	1 (33.3)		14 (93.3)
Alanine aminotransferase increased	1 (33.3)		1 (33.3)			1 (11.1)	7 (77.8)	1 (11.1)			1 (33.3)	2 (66.7)	14 (93.3)
Aspartate aminotransferase increased	1 (33.3)		1 (33.3)			3 (33.3)	5 (55.6)				1 (33.3)	2 (66.7)	13 (86.7)
Neutrophil count decreased		1 (33.3)	1 (33.3)				2 (22.2)	6 (66.7)		1 (33.3)		2 (66.7)	13 (86.7)
Constipation	2 (66.7)				6 (66.7)				2 (66.7)	1 (33.3)			11 (73.3)
Vomiting	2 (66.7)				6 (66.7)				2 (66.7)				10 (66.7)
White blood cell count decreased						1 (11.1)	4 (44.4)	2 (22.2)		1 (33.3)		2 (66.7)	10 (66.7)
Malaise	1 (33.3)				4 (44.4)		1 (11.1)		1 (33.3)	2 (66.7)			9 (60.0)
Gamma-glutamyltransferase increased					2 (22.2)	1 (11.1)	3 (33.3)			1 (33.3)	2 (66.7)		9 (60.0)
Decreased appetite	1 (33.3)				4 (44.4)		1 (11.1)			1 (33.3)	2 (66.7)		9 (60.0)
Lymphocyte count decreased					1 (11.1)	1 (11.1)	1 (11.1)	2 (22.2)		2 (66.7)		1 (33.3)	8 (53.3)
Anaemia					2 (22.2)	1 (11.1)	1 (11.1)	1 (11.1)			1 (33.3)	1 (33.3)	7 (46.7)
Platelet count decreased					1 (11.1)		1 (11.1)	2 (22.2)			1 (33.3)	2 (66.7)	7 (46.7)
Blood creatine phosphokinase increased	1 (33.3)				2 (22.2)			1 (11.1)			1 (33.3)	1 (33.3)	6 (40.0)
Pyrexia	1 (33.3)				2 (22.2)				1 (33.3)	1 (33.3)			5 (33.3)
Myalgia					2 (22.2)				2 (66.7)				4 (26.7)
Headache					4 (44.4)								4 (26.7)
Blood creatinine increased					1 (11.1)				1 (33.3)	1 (33.3)			3 (20.0)
Electrocardiogram QT prolonged					2 (22.2)						1 (33.3)		3 (20.0)
Blood alkaline phosphatase increased					2 (22.2)				1 (33.3)				3 (20.0)
Hyperkalaemia		1 (33.3)			1 (11.1)				1 (33.3)				3 (20.0)
Hypokalaemia					1 (11.1)					1 (33.3)	1 (33.3)		3 (20.0)

Analysis Set: All Treated Patients

If a patient is reported to have the same toxicity more than once, then that patient is only counted once for the summary of that toxicity, using the most severe intensity

^a^Incidence Rate(%) = (Number of patients experienced adverse events in each Preferred Term)/(Number of patients in each dosage level) × 100

A total 13 serious AEs occurred in six patients (1 of 3 patients at dose level 1, 2 of 9 at dose level 2, and 3 of 3 at dose level 3). Platelet count decreased and anorexia developed in two or more patients. All serious AEs were attributed to trabectedin and resolved after appropriate treatment. There was no treatment-related death during the study. One patient at dose level 2 and 2 patients at dose level 3 withdrew from the study because of AEs related to trabectedin (neutrophil count decreased, rhabdomyolysis, and platelet count decreased, respectively).

### Pharmacokinetics

**Fig. 1:**
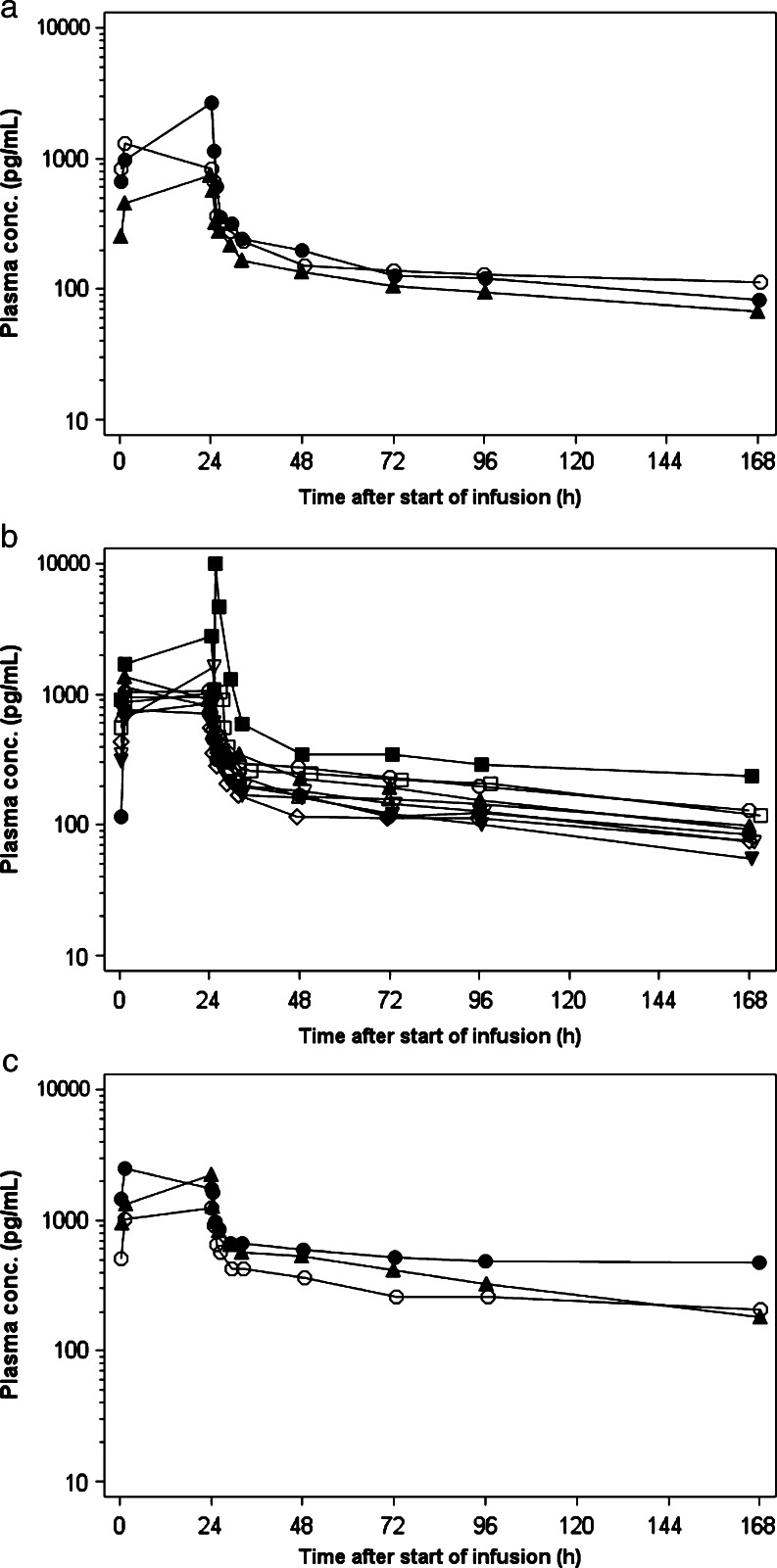
Individual plasma concentration time profiles of trabectedin for **a** dose level 1, **b** dose level 2 and **c** dose level 3

Individual plasma concentrations of trabectedin are shown in Fig. 1, and pharmacokinetic variables are shown in Table 3. Plasma trabectedin concentrations decreased immediately after the end of infusion, and the drug was gradually eliminated. Mean AUCs increased in a dose-dependent manner. In one patient given dose level 2, the plasma trabectedin concentration abnormally rose again up to 9,900 pg/mL 1 h after the completion of infusion. Elimination at dose level 3 was slower than that at the lower dose levels.

**Table 3:** Pharmacokinetic parameters of trabectedin

		Level 1 (*N* = 3)			Level 2 (*N* = 9)			Level 3 (*N* = 3)			
		Mean	SD	CV (%)	Mean	SD	CV (%)	Mean	SD	CV (%)	n
t_max_	(h)	16.5	13.0	78.9	17.2	11.8	68.7	16.6	13.1	78.7	3
C_max_	(pg/mL)	1570	997	63.3	2070	2950	142.0	1990	652	32.8	3
λz	(1/h)	0.0037	0.0016	42.5	0.0060	0.0015	25.3	0.0056	NC	NC	2
t_1/2_	(h)	221	126	57.2	124	35	28.5	174	NC	NC	2
AUC_0–48_	(ng·h/mL)	33.3	15.8	47.5	35.3	18.5	52.4	53.5	14.9	27.8	3
AUC_0-t_	(ng·h/mL)	46.9	17.3	36.8	53.9	25.3	46.8	96.3	29.0	30.1	3
AUC_inf_	(ng·h/mL)	77.7	31.8	40.9	74.9	42.7	56.9	132	NC	NC	2
CL	(L/h/m^2^)	13.3	6.4	47.9	18.7	5.8	31.0	11.6	NC	NC	2
Vd_ss_	(L/m^2^)	2470	1210	48.8	2030	716	35.4	2110	NC	NC	2
V_z_	(L/m^2^)	3710	1170	31.6	3160	989	31.3	2710	NC	NC	2
CL	(L/h)	21.0	11.7	55.6	31.4	9.5	30.1	21.1	NC	NC	2
Vd_ss_	(L)	3790	1810	47.6	3380	1050	31.0	3800	NC	NC	2
V_z_	(L)	5730	1830	32.0	5290	1420	26.8	4900	NC	NC	2

*SD* standard deviation, *CV* coefficients of variation

### Patient responses

The maximum number of administered treatment cycles was 7, 19, and 2 at dose level 1, 2 and 3, respectively. In the 15 patients, the response rate was 20 % (3 of 15) and the progression-free rate (PFR) at 3 months was 60 % (9 of 15). All three patients with partial response (PR) were at dose level 2 and had translocation-related sarcomas (TRS; myxoid liposarcoma, synovial sarcoma, extraskeletal Ewing sarcoma in one patient each) with confirmation of fusion genes (EWS-CHOP1, SYT-SSX1, and EWS mutation, respectively).

**Fig. 2:**
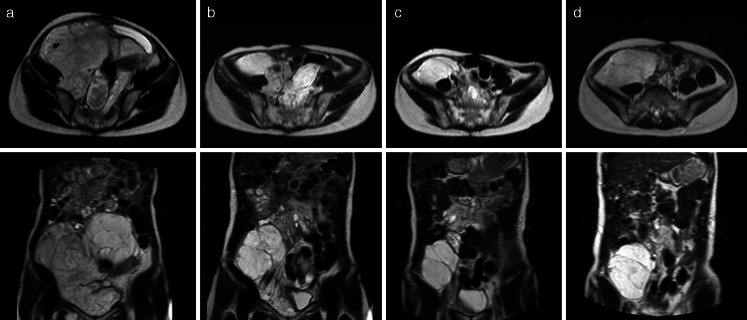
MRI findings of a 21-year-old female patient with a recurrent retroperitoneal myxoid liposarcoma: **a** baseline, and after **b** 7 cycles (day 232), **c** 13 cycles (day 421) and **d** 19 cycles (day 672) of trabectedin at dose level 2 (1.2 mg/m^2^)

The case of a patient who continues the longest treatment with trabectedin is presented. A 13-year-old girl was given a diagnosis of a retroperitoneal myxoid liposarcoma in 2004. She received three lines of chemotherapy, including pirarubicin, ifosfamide, and dacarbazine, and then underwent surgery to remove the entire tumor from the pelvis. She had relapse 2 years later, underwent surgery six times, and received topotecan and vaccine therapy for advanced disease over the course of 5 years. When she was 21 years of age, she presented at a site participating in this study. The patient started to receive trabectedin at dose level 2 in September 2011. Magnetic resonance imaging (MRI) showed a retroperitoneal lesion (longest diameter, 249 mm; Fig. 2a) at baseline. MRI showed a 33 % reduction in tumor diameter (167 mm, Fig. 2b) at 7 cycles and the shortest diameter was 83 mm at 13 cycles (Fig. 2c). The latest diameter was 122 mm at 19 cycles (Fig. 2d), which meet the criteria of progressive disease (PD.

## Discussion

This phase I study of trabectedin in Japanese patients with advanced STS indicated that the MTD is 1.5 mg/m^2^ and that the recommended dose for phase II clinical trials is 1.2 mg/m^2^ in Japan.

Trabectedin 1.5 mg/m^2^ as 24-h infusion every 3 weeks is approved by the European Medicines Agency (EMA) for STS based on the results of a phase I study in patients with solid tumors [18] and a phase II study in patients with liposarcoma or leiomyosarcoma, which showed that trabectedin 1.5 mg/m^2^ every 3 weeks was associated with a longer time to progression than 0.58 mg/m^2^ every week [22].

In our study, two of three patients at dose level 3 had DLT, and the third patient had rhabdomyolysis and withdrew. All three patients also had serious AEs at dose level 3. At dose level 2, no patient had DLT. Only one patient withdrew from the study because of trabectedin-related neutrophil count decreased.

The incidence of adverse drug reactions increased in parallel to the dose of trabectedin. The number of grade 3 and 4 events including increase in hepatic transaminase and CPK was higher at dose level 3 than at dose level 1 or 2. Based on our safety data, we estimated that 1.2 mg/m^2^ trabectedin can be administered safely to Japanese patients with STS. Thus, the present study suggested that we warn toxic expression and consider starting trabectedin treatment at 1.2 mg/m^2^ for Asian patients.

Plasma trabectedin concentrations promptly decreased after the completion of infusion, and the drug was gradually eliminated. The cause of the abnormally high drug concentration in a patient at dose level 2 is unclear, but the data were included in analysis. Owing to this high plasma concentration of trabectedin, the coefficients of variation for C_max_, AUC_0-t_, and AUC_inf_ were around 50 %, which indicated wide variability at dose level 2. At dose level 3, high plasma trabectedin concentrations persisted after the completion of infusion in two patients who had DLTs and severe elevations of AST and ALT. Mean AUC_0-t_ and AUC_inf_ were slightly higher than the respective values at the other dose levels, although C_max_ was similar. The fact that two patients had severe AEs at dose level 3 may indicate a relation between pharmacokinetics and toxicity.

**Fig. 3:**
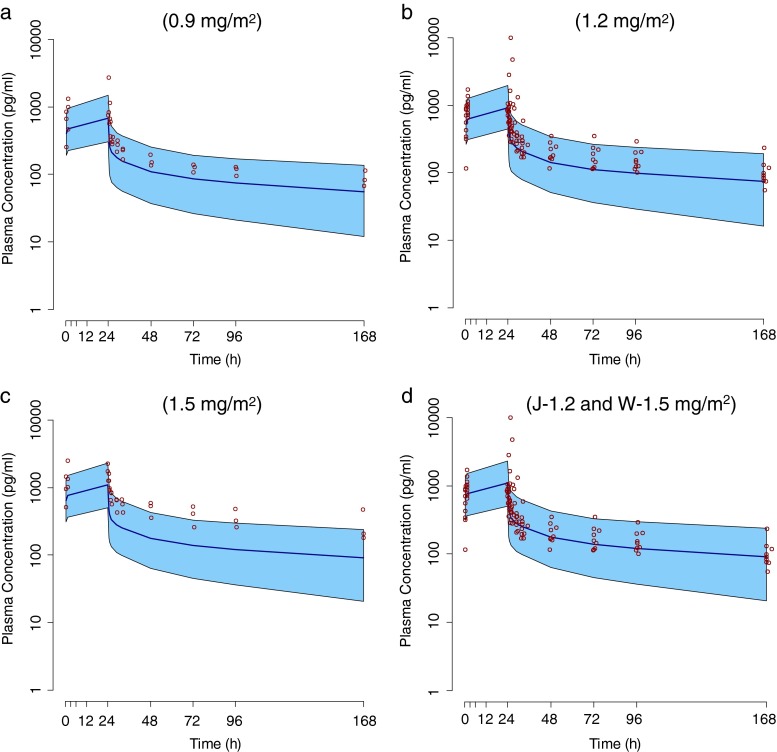
Individual plasma concentrations of Japanese patients (*red circle*) over VPC 90 % (blue area; data from Caucasian PopPK model at the same dose level) (25) for **a** dose level 1, **b** dose level 2 and **c** dose level 3. **d** Individual plasma concentrations of Japanese patients at dose level 2 (J-1.2) over VPC 90 % of Caucasian Pop PK model at 1.5 mg/m^2^ (W-1.5)

CL and Vd_ss_ in our study were lower than the reported values (CL: 51.44 L/h and Vdss: 4981 L) in a previous phase II study in Caucasian patients with STS (PharmaMar SA, 2006). We compared our data with the reported PopPK model data in Caucasian patients [19] using visual predictive checks (VPC). Plasma concentrations in Japanese patients were slightly higher than that of the population mean estimated by the PopPK model for each dose level, and at higher dose level, several plasma concentrations were higher than the upper range of VPC (Fig. 3a–c). Plasma concentrations at dose level 2 in our study seemed to correspond to plasma concentrations at 1.5 mg/m^2^ trabectedin in Caucasian patients (Fig. 3d). Because the clearance of trabectedin correlated with clearance of midazolam, it is apparent that the clearance of trabectedin depends on the hepatic clearance by CYP3A4 which is a main metabolism enzyme for trabectedin [23]. It is reported that there is not ethnic difference over individual difference in pharmacokinetics of midazolam [24]. Currently, the reason of the difference between Japanese and Western patients in the clearance of trabectedin is unclear.

As for the efficacy of trabectedin, the overall response rate of 20 % was higher than the response rate obtained in a retrospective analysis of phase II studies of trabectedin in patients with advanced STS (7 % among 620 patients) [25]. The PFR of 60 % at 3 months also supported further investigation of this agent for STS [26]. Encouraging disease control by trabectedin was expected especially in TRS as reported previously [27].

In conclusion, recommended dose of trabectedin for phase II clinical trials was 1.2 mg/m^2^ in Japanese patients with advanced STS refractory to anthracyclines. A randomized phase II study comparing 1.2 mg/m^2^ trabectedin with best supportive care is ongoing in Japanese patients with TRS. Further pharmacokinetic evaluations of trabectedin are scheduled to be performed in this phase II study to examine the reasons for the difference in the recommended dose between Japanese and Western patients.
